# Seroepidemiology of Human Parvovirus B19 Infection among the Population of Vojvodina, Serbia, over a 16-Year Period (2008–2023)

**DOI:** 10.3390/v16020180

**Published:** 2024-01-25

**Authors:** Vladimir Vuković, Aleksandra Patić, Mioljub Ristić, Gordana Kovačević, Ivana Hrnjaković Cvjetković, Vladimir Petrović

**Affiliations:** 1 Institute of Public Health of Vojvodina, 21000 Novi Sad, Serbia; aleksandra.patic@mf.uns.ac.rs (A.P.); mioljub.ristic@mf.uns.ac.rs (M.R.); gordana.kovacevic@izjzv.org.rs (G.K.); ivana.hrnjakovic@izjzv.org.rs (I.H.C.); vladimir.petrovic@izjzv.org.rs (V.P.); 2 Department of Epidemiology, Faculty of Medicine, University of Novi Sad, 21000 Novi Sad, Serbia; 3 Department of Microbiology with Parasitology and Immunology, Faculty of Medicine, University of Novi Sad, 21000 Novi Sad, Serbia

**Keywords:** human parvovirus B19, PVB19, seroprevalence, general population, pregnancy, Serbia

## Abstract

This study aimed to estimate the serological status and dynamic changes in the prevalence of Parvovirus B19 (PVB19) antibodies within the general population residing in the northern part of the Republic of Serbia (Province of Vojvodina) during a 16-year period. Serum samples were analyzed for Human PVB19-specific IgM and IgG antibodies using enzyme-linked immunosorbent assay (ELISA). Throughout the study period, the overall seroprevalence was 49.51%. Approximately 10% of patients exhibited a serologic profile positive for PVB19 IgM antibodies. Notably, seroprevalence varied significantly, ranging from 9.12% in the pediatric cohort (ages 1–4 years) to 65.50% in the adult demographic (40–59 years old). Seroprevalence was higher (51.88%) among women compared to men (42.50%). Immunologically naive pregnant women in the age groups 26–36 and 36–45 years had 45% (OR = 0.55, 95% CI: 0.31–1.00) and 52% (OR = 0.48; 95% CI: 0.24–0.94) lower odds of having negative IgM and IgG compared to those in age group 16–25 years old. Improved knowledge of the epidemiology of PVB19 may assist clinicians in the differential diagnosis of PVB19 clinical manifestations. The PVB19 detection is particularly important for monitoring individuals in risk groups such as women of reproductive age, medical staff, patients with hematological disorders, and those with immunodeficiency.

## 1. Introduction

Parvovirus B19 (PVB19) is a small, non-enveloped single-stranded DNA virus from the family of Parvoviridae that causes infection in humans, usually entering through the respiratory tract [1,2], by blood transfusion, bone marrow transplantation, or vertically from mother to the fetus during pregnancy [3]. It has tropism for erythroid progenitor cells, causing the suppression of erythropoiesis due to its cytotoxic effect [4].

PVB19 infection has been associated with a spectrum of clinical manifestations, depending on immunological competence and the age of patients, from asymptomatic and subclinical infections to erythema infectiosum, arthropathy, central and peripheral neurologic manifestations, chronic anemia, and transient aplastic crisis [4,5,6]. In children, PVB19 infection is most commonly presented as a febrile disease with a characteristic skin rash, i.e., erythema infectiosum (so-called fifth disease), while during the adulthood, it is most frequently presented as unspecific fever. In more severe cases, it can progress to acute arthritis, arthropathy, transient aplastic crisis, and hemolytic anemia [1]. In patients with underlining hematological disorders, like spherocytosis, sickle cell, and hemolytic anemia, infection with PVB19 may lead to aplastic anemia crisis [7,8]. Additionally, in immunocompromised patients, PVB19 can lead to prolonged anemia, myocarditis, hepatitis, and pneumonia [9,10]. Infection with PVB19 in pregnancy, even though it has no teratogenic effects, may lead to severe outcomes like spontaneous abortion, fetal hydrops, or intrauterine fetal death [11,12,13,14] in approximately 10% of infected fetuses, especially when it happens in the first two trimesters of pregnancy [15]. The estimated risk of transplacental infection is up to 33% [16].

PVB19 outbreaks occur every 3–5 years, mostly during the winter and spring periods [17,18], and are common in schools and kindergartens due to a relatively easy spreading of the virus [19]. Humoral immunity has a major (leading) role in controlling the PVB19 infection, since it was demonstrated to provide a lifelong protection against re-infection [1,4]. Detection of PVB19-specific antibodies is essential for a proper diagnosis of infection, while in immunocompromised patients, the detection of PVB19 DNA is usually required [20].

Seroepidemiological data of PVB19 infection demonstrate considerable differences worldwide, with more than half of adults being exposed at some point during their lifetime [1]. It is an endemic infection in practically all parts of the world, and the exposure to the virus is quite frequent; however, due to a high prevalence of asymptomatic cases, seroconversion usually happens without a manifested disease [1]. Different national and international seroepidemiological studies have demonstrated heterogeneous results, with a commonly reported increase of seroprevalence with age [21,22,23,24,25]. Seroprevalence rates from China, Japan, and five European countries, depending on the immunological assay and population under study, ranged from 2–20% in the youngest group of children (under the age of 5), followed by 15–60% among children of 5–19 years old, and 40–80% in the adults, reaching up to 85% of seropositivity in the elderly group [18,26,27,28]. Of particular interest is the seroprevalence in women of reproductive age, where almost half of women of childbearing age are susceptible to PVB19 infection, with potentially even higher percentages in developing countries [1,13,29,30].

Currently, there is no effective vaccine against PVB19 available even though few candidates are under evaluation with some promising results [31,32]. Precise seroepidemiological data about the frequency of PVB19 infections are crucial in order to properly respond to the challenges when dealing with prevention strategies for these infections [33], and in particular, in susceptible and vulnerable populations, like pregnant women and the immunocompromised. In Serbia, as in many European countries, PVB19 infection is not a notifiable disease, and since it is often presented as asymptomatic, it remains difficult to have a comprehensive overview of its true prevalence. There is no up-to-date comprehensive study examining PVB19 seroprevalence conducted in Serbia; thus, we took the aim of analyzing the characteristics of the population groups most commonly tested for PVB19 to investigate the seasonality of acute PVB19 infection and to identify age group at the highest risk for developing parvovirus infection within the general population of the Province of Vojvodina (northern part of Serbia). Additionally, the trend in seroprevalence of anti-PVB19 IgG antibodies during 16 years under surveillance was determined.

## 2. Materials and Methods

### 2.1. Study Area

Vojvodina, an autonomous province situated in the northern region of the Republic of Serbia (coordinates: 45°30′12.92″ north latitude and 20°3′2.84″ east longitude), spans a total surface area of 21,500 km^2^ (8300 sq. mi). Notably, nearly one-third of Serbia’s population (excluding autonomous province of Kosovo and Metohija) resides in the province of Vojvodina [34].

### 2.2. Study Population and Data Collection

This cross-sectional study involved a cohort of 8692 individuals residing in Vojvodina, Serbia. The investigation, conducted between 1 September 2008 and 1 September 2023, included both outpatients from Government Healthcare Centers across Vojvodina and inpatients from the Clinical Center of Vojvodina. Serum samples from these participants were sent to the Center of Virology, Institute of Public Health of Vojvodina (IPHV), for the performance of serological tests targeting Human PVB19. The indications for serological testing were linked to diverse medical diagnoses and conditions associated with this pathogen. Data obtained from the Protocol of Virology Analyses at the Center of Virology (IPHV) encompassed participant information on age, sex, and different medical conditions. Venous blood samples (3 mL) were collected in EDTA-free tubes following established operational procedures. Post-centrifugation, the serum was transferred into clean polypropylene tubes and stored at 4–8 °C until analysis. Adhering to our laboratory procedures, diagnostic methods were executed within 1–3 days.

### 2.3. Laboratory Method

Serum samples underwent analysis for PVB19-specific immunoglobulin M (IgM) and G (IgG) antibodies through enzyme-linked immunosorbent assay (ELISA), utilizing commercial Euroimmun (Lübeck, Germany) and VirionSerion (Würzburg, Germany) kits. All procedures were automated, following the manufacturer’s instructions for use. Both IgG and IgM results were semiquantitatively evaluated by calculating the ratio of the extinction value of the control or patient sample over the extinction value of the appropriate calibrator. Interpretation of IgG and IgM antibody ratios adhered to the manufacturer’s guidelines. For samples tested with VirionSerion, results were interpreted based on the cut-off levels in accordance with the manufacturer’s recommendations: as negative if IgM < 10 or IgG < 3, equivalent if IgM = 10–15 or IgG = 3–5, or positive if IgM > 15 or IgG > 5, for the presence of IgM and IgG antibodies in serum samples. Similarly, results of the samples tested with Euroimmun were interpreted as negative if IgM or IgG were <0.8, equivalent if in the range of 0.8–1.1, or positive if >1.1. The management of borderline results involved the following steps: (a) PVB19 IgM negative results were assigned if IgG seroconversion was not detected or if there was not a significant IgG titer change in the second sample following the initial IgM borderline and IgG-negative result; (b) patients were excluded from the study if the first result was borderline for PVB19 IgM and in situations when the second specimen was not available; (c) samples with repeated equivocal IgM or IgG results requiring further analysis were excluded from clinical interpretation. Overall, patients with ELISA borderline results, for whom diagnosis could not be resolved, were excluded from the study.

### 2.4. Statistical Analyses

Descriptive statistics with absolute frequencies and percentages (%) with 95% confidence intervals (95% CI) were used to present data for categorical variables. Based on patients’ age, we constructed general age categories (0–4, 5–9, 10–19, 20–39, 40–59, and ≥60 years old), as well as children’s and adolescent’s age categories (<1, 1–3, 4–6, 7–14, and 15–19 years old). Additionally, based on age, we grouped patients into three more broad categories: children and adolescents (0–19 years old), adults (20–64), and elderly (≥65 years old). Finally, for women of generative age (14–45 years old), we considered their referral diagnosis and divided them into two categories: pregnant and non-pregnant women, in order to explore the differences in their serological status. Results from women of generative age were also analyzed based on their age category (14–25, 26–35, and 36–45 years old).

Seroprevalence of PVB19 IgM and IgG was calculated as the percentage of test-positive patients among the total number of tested patients, respectively. Finally, seroprevalence of PVB19 was calculated separately for patients referred under certain diagnoses specified when samples were delivered to the IPHV (i.e., fever; urticaria; rash; erythema; erythema infectiosum; erythema any; myocarditis; arthritis/arthralgia; transplantation; miscarriage; infertility) due to the particular significance of PVB19 infection in their etiology. Additionally, patients were grouped into three categories based on the serological result: as having acute/recent infection, when PVB19 IgM was positive; as being immune (past infection), if IgM was negative and IgG was positive; and naïve (not immune), when both, IgM and IgG were negative. Finally, seasons, when serological testing was conducted and diagnosis were made, were defined using definition of the meteorological seasons for the northern hemisphere, which is based on the annual temperature cycle (i.e., spring includes March, April, and May; summer includes June, July, and August; autumn includes September, October, and November; and winter includes December, January, and February of each included calendar year). The seasons begin on the first day and end on the last day of the month [35].

The chi-squared test was used to test differences in distributions between different groups, and the Pearson correlation analysis was used to explore the correlation between patients’ age and distribution of patients by immunological status. Additionally, we used the logistic regression model to explore predictive factors associated with PVB19 acute infection status. Univariate analysis was conducted using independent variables: sex, age (continuous and categorical: children and adolescents, adults, elderly), pregnancy, and season when testing was conducted. Based on the results of univariate analysis, age and sex were used for adjusting in the multivariate analyses. The odds ratio (OR) with the corresponding 95% CI was calculated to demonstrate the strength of association. All statistical analyses were performed using Stata v.17 (STATA StataCorp, College Station, TX, USA), and results at the *p*-value < 0.05 were considered statistically significant across the analyses.

### 2.5. Ethical Approval

Data from routine laboratory diagnostics of outpatient and inpatient care facilities, or after the recommendation for laboratory testing by attending physicians, were used. In accordance with applicable laws and regulations, no informed consent was needed for the retrospective analysis of anonymized data, collected as part of routine health analyses. The study was conducted in accordance with the Declaration of Helsinki and was approved by the Institutional Ethical Committee Board of the Institute of Public Health of Vojvodina, Novi Sad, Serbia (protocol number: 01-1589/2, date of approval: 13 November 2023). The authors were not involved in the treatment or clinical follow-up of the included patients, and all data with personal information were anonymized before being accessed and analyzed by the authors.

## 3. Results

During the period under surveillance, from September 2008 to September 2023, a total of 8692 samples were analyzed for the presence PVB19 IgM, 7325 for PVB19 IgG antibodies, while 7255 samples were tested for both PVB19 IgM and IgG. There were 753 (8.66%; 95% CI: 8.08–9.27) samples with positive PVB19 IgM, and a total of 3529 (48.18%; 95% CI: 47.03–49.33) samples with positive PVB19 IgG antibodies. The majority of samples were analyzed using the Euroimmun ELISA test, i.e., 54.48% and 59.60% of the samples tested for the presence of IgM and IgG, respectively.

Among 5215 tested samples from females and 3477 from males, 9.80% (95% CI: 9.00–10.64) and 6.96% (95% CI: 6.14–7.86) were positive for the presence of PVB19 IgM in serum, respectively. The highest percentage of positive samples was reported in the group of 10–19 years old (10.52%; 95% CI: 8.93–12.29), followed by 5–9 years old (9.99%; 95% CI: 7.89–12.41) and 20–39 years old (9.30%; 95% CI: 8.26–10.42). The majority of the samples (4492/8692, 51.68%) arrived from hospitalized patients, but there was a higher percentage of positive PVB19 IgM among outpatients, 9.26% (95% CI: 8.40–10.18), with respect to hospitalized patients (8.10%; 95% CI: 7.32–8.94) (*p* = 0.055). Outpatients aged 20–39 years had a significantly higher (*p* = 0.023) percentage of PVB19 IgM-positive results (10.16%; 95% CI: 8.85–11.60) than inpatients in the same age group (7.52%; 95% CI: 5.91–9.40). General characteristics of the study population are presented in more detail in Table 1.

On the other hand, 51.88% (95% CI: 50.41–53.36) of tested samples from females and 42.50% (95% CI: 40.69–44.32) from males were positive for the presence of PVB19 IgG (Table 2). The highest percent of positive samples was from the group of 40–59 years old (65.48%; 95% CI: 62.92–67.97), followed by those of ≥60 years old (61.51%; 95% CI: 57.47–65.44). The majority of samples were from ambulatory patients (3995/7325, 54.54%), and the seroprevalence of PVB19 IgG was significantly (*p* < 0.001) higher among outpatients (54.94%; 95% CI: 53.39–56.49) than in the hospitalized patients (40.06%; 95% CI: 38.39–41.75). More precisely, outpatients regardless of sex and those aged 10–19 years had significantly higher seroprevalence of PVB19 IgG in comparison with the same group among hospitalized patients.

**Figure 1:**
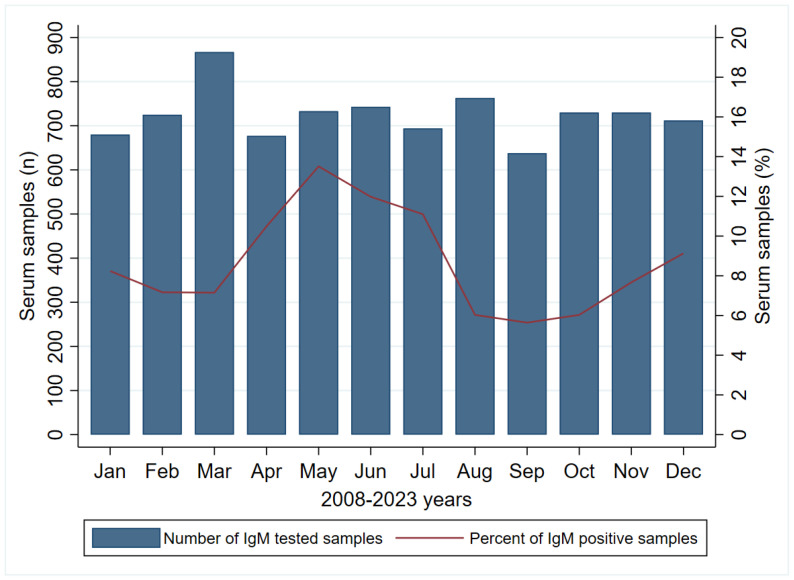
Number of tested samples and percent of positive PVB19 IgM samples by month of registration in Vojvodina, Serbia, 2008–2023.

Across the period under surveillance (2008–2023), when observing the cumulative number, the highest number of samples tested for the presence of IgM was in the following months: March (n = 867; 9.97%), August (n = 763; 8.78%), and June (n = 743; 8.55%), while the highest percentage of positive samples, 13.51%, 11.98%, and 11.10%, were reported in May, June, and July, respectively, as presented in Figure 1.

**Figure 2:**
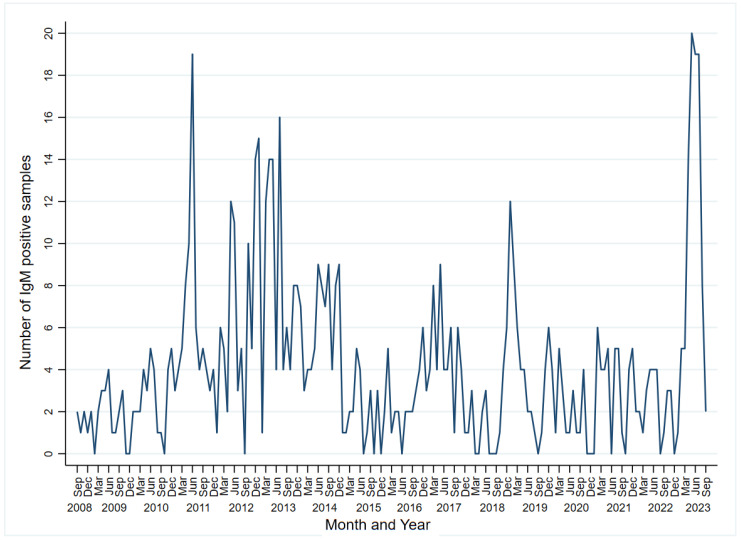
Monthly series of PVB19-specific IgM-positive samples in Vojvodina, Serbia, 2008–2023.

Specifically, the highest number of samples tested positive for the presence of IgM was in 2023 in the following months: May 2023 (n = 20, 18.87%), June (n = 19, 18.27%), and July (n = 19, 20.43%), as presented in Figure 2. The average number of cases diagnosed per month was 4.16 (SD = 4.03).

The seasonal pattern of IgM positivity was also reported across the 2008–2023 years (Figure S1), with the higher percentage of positive samples during the spring (10.19%) and summer (9.64%) periods, with respect to winter (8.17%) and autumn (6.48%) (*p* < 0.001).

Also, when analyzing a yearly distribution of positive IgM samples, we noticed that the highest percentage was reported in the year 2011 (18.70%), followed by 2012 (17.10%) and 2013 (15.40%) (Figure S2).

When analyzing the seroprevalence of PVB19 IgM according to age category, we found that the highest prevalence was in children and adolescents (10.22%), followed by adults (8.80%) and the elderly (4.91%) (*p* = 0.001). Regarding sex, the higher percentages of IgM seropositivity, in both females and males, were in the group of children and adolescents, 12.14% and 8.62%, respectively. Regarding seroprevalence of PVB19 IgG, the highest percentage was in the group of the elderly (67.27%), followed by adults (62.66%) and children and adolescents (27.08%) (*p* < 0.001), with a total of 53.25% of samples positive for IgG in females and 43.62% in males (Table 3).

Serological results and the prevalence of PVB19 infection in 6743 samples of 7255 tested for both, IgM and IgG, and after removing 512 samples with equivalent results (i.e., 337 equivalent for IgM, 170 equivalent for IgG, and 5 equivalent for both Ig), are presented in Table 4. We noticed that 3.66% (95% CI: 3.23–4.14) of patients had positive IgM and negative IgG, suggesting acute/recent PVB19 infection, while 5.43% (95% CI: 4.90–5.99) had positive IgM and IgG, indicating PVB19 infection in the recent period (last 7–120 days). Past infection with PVB19 (negative IgM and positive IgG) was observed in 43.63% (95% CI: 42.44–44.82) of patients, and the frequency of naïve patients (negative IgM and IgG) was 47.28% (95% CI: 46.08–48.48).

Specifically, acute PVB19 infection was present in 11.95% of children and adolescents, 10.59% of adults, and 6.95% of the elderly (Table 5). There was a statistically significant difference between males and females across these age categories, with the majority of children and adolescents being naïve, i.e., 67.94% of boys and 63.07% of girls, while in the groups of adults and the elderly, the majority were immune (more than 50% across all categories), indicating past infection with PVB19 (*p* < 0.001).

**Figure 3:**
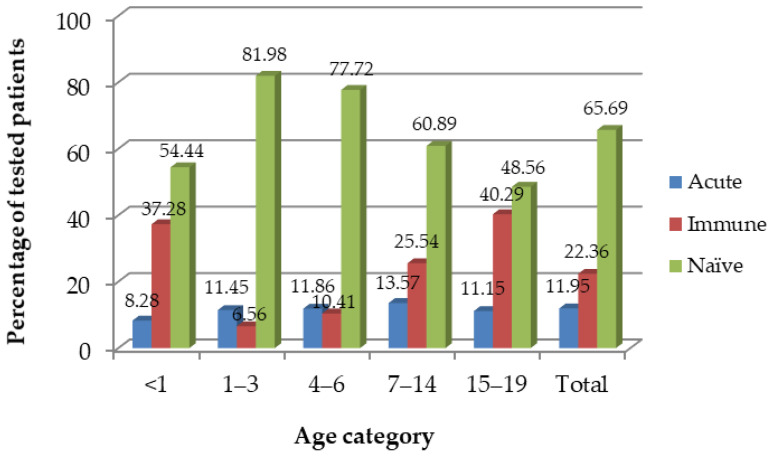
Prevalence of IgM-positive (acute), IgG-positive (immune), and IgM/IgG-negative (naïve) PVB19 immunological status by age category in the sample of children and adolescents, Vojvodina, Serbia, 2008–2023. Note: a high proportion of immune infants (<1 years old) might reflect IgGs passing transplacentary from their mothers and not necessarily infants’ past PVB19 infection and thus should be interpreted with caution.

Stratified analyzes for children and adolescents by age and their immunological status are presented in Figure 3. The majority of patients in this age group (0–19 years old) were immunologically naïve (65.69%), followed by 22.36% of those immune, and 11.95% of patients in the acute phase of PVB19 infection. Specifically, around 13.60% of children aged 7–14 years old had acute infection, as well as 11.90% of kids aged 4–6 years and 11.45% of children 1–3 years old.

Additionally, we analyzed immunological status of 411 pregnant women that were referred for IgM/IgG testing by their age (Table 6). The highest percent were immune (53.04%) at the time of testing, followed by 35.74% of immunologically naïve, and 10.22% with acute PVB19 infection. In particular, those of younger age (14–25 years old) were predominately naïve (50%), while those 26–35 and 36–45 years old were predominantly immune, with 53.64% and 55.21% of tested pregnant women, respectively, being in these categories. Pregnant women aged 26–35 had 45% (OR = 0.55, 95% CI: 0.31–1.00), and those of 36–45 years had 52% (OR = 0.48; 95% CI: 0.24–0.94) lower odds of having negative IgM and IgG (being immunologically naïve) results with respect to those in the age group of 14–25 years old.

Similarly, when analyzing the immunological status of non-pregnant women of generative period (14–45 years old), we noticed the highest percent of immune population (51.12%) overall, with 55.91% of those 36–45 years old and 52.55% of those 26–35 years old in this category (Table 7). Also, older non-pregnant women in the generative period had lower odds of being immunologically naïve (IgM/IgG-negative) in respect to the youngest category (i.e., 36–45 years old, OR = 0.57, 95% CI: 0.45–0.71; and 26–35 years old, OR = 0.67, 95% CI: 0.53–0.84).

**Figure 4:**
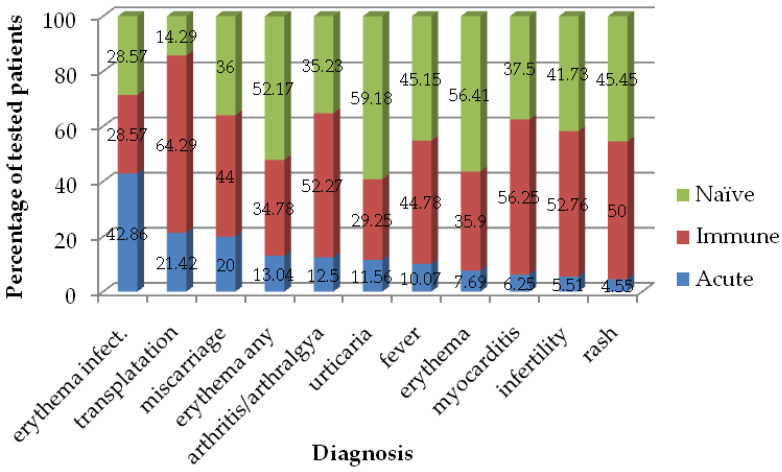
Seroprevalence of IgM-positive (acute), IgG-positive (immune), and IgM/IgG-negative (naïve) PVB19 in patients referred under certain diagnoses in Vojvodina, Serbia, 2008–2023.

In Figure 4, the immunological statuses of patients with certain referred diagnosis are presented. We observed the highest percent of acute patients (42.86%) in the group of those with referred diagnosis erythema infectiosum, followed by those in the group of transplanted patients (21.42%), and those with miscarriage (20%).

Results from uni- and multi-variate logistic regression analysis, exploring factors associated with the presence of acute PVB19 infection, are presented in Table 8. We noticed that females had higher odds of having acute PVB19 infection (OR = 1.44; 95% CI: 1.22–1.69) with respect to males. At each year increase of patient’s age, the odds of having acute infection decreased by 1% (OR = 0.99; 95% CI: 0.99–0.99). Specifically, the elderly had 45% lower odds of having acute infection (OR = 0.55; 95% CI: 0.35–0.85) with respect to children and adults. Finally, those tested during autumn demonstrated lower odds of having acute infection (OR = 0.61; 95% CI: 0.49–0.76).

**Figure 5:**
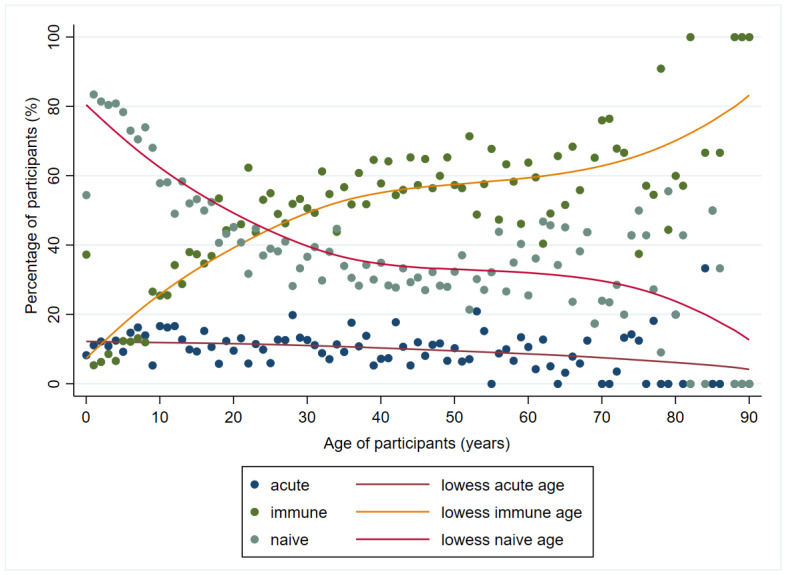
Proportion of patients by immunological status and by age in Vojvodina, Serbia, 2008–2023. Note: lowess = locally weighted scatterplot smoothing.

Finally, when exploring correlations between patients’ age and the distribution of patients by immunological status, we noticed a negative correlation for those with acute (r = −0.35, *p* < 0.001) and naïve (r = −0.70, *p* < 0.001), and a positive for those immune (r = 0.75, *p* < 0.001), demonstrating the fact that when the age increases, the percentage of immune in the population is increasing and the percent of naïve is decreasing (Figure 5).

## 4. Discussion

The present study was performed to investigate the temporal dynamics of anti-PVB19 antibodies (IgM and IgG) prevalence in the general population of the Province of Vojvodina, the northern region of the Republic of Serbia. The study spanned 16 consecutive years, revealing an overall seroprevalence of IgG antibodies, i.e., an earlier infection with PVB19, in 49.51% of the patients. Notably, seroprevalence varied significantly, ranging from 9.12% in the pediatric cohort (ages 1–4 years) to 65.50% in the adult (40–59 years old) population. Previous seroprevalence studies have indicated global circulation of human parvovirus B19V without clearly discernible ethnic or geographic boundaries, although with considerable heterogeneity among countries and regions [26]. Our results showed a slightly lower seroprevalence compared to other European countries, with reported seroprevalence in Poland (52.90%) [36], England (53%) [37], Germany (72.10%) [18], Croatia (64.10%) [10], and the Netherlands (61%) [23]. Although the acquisition pattern of PVB19 infection remains consistent, variations in seroprevalence among diverse populations may be ascribed to differences in age distribution within the study design and variations in serological techniques employed to assess parvovirus exposure. One contributing factor to the relatively lower seroprevalence in our study may be the inclusion of nearly one-third of the examined population comprising children under 18 years old. Importantly, it is pertinent to acknowledge that seroprevalence data for PVB19 in some European countries are over a decade old and necessitate updating.

In our study, the highest percentage of positive samples was found in the group of 40–59-year-old patients (65.48%), followed by the ≥60-year-old patients (61.51%). Research carried out in five European countries (Belgium, Finland, Italy, Poland, England, and Wales) showed that the percentage of people with protective IgG antibodies increased with the age of the subjects and ranged from 5 to 40% at the age of 1–9 years to 40–63% at the age of 10–18 years [26], which is in line with our results. Notably, a high percent of IgG positive samples (41.53%) in the youngest group of patients (<1 years) in our study most likely reflects the transplacentary passed antibodies that still circulated in their blood.

Albeit positive IgM results were detected in all age categories of Vojvodina residents, the highest percentage was reported in the group of 10–19year-old patients (10.52%), followed by 5–9-year-old (9.99%) and 20–39-year-old patients (9.30%). These results in line with the fact that infection by PVB19 more frequently spreads among children in kindergartens, schools, and boarding schools. Results of the studies given by other authors have also demonstrated that the frequency of acute PVB19 infections is the highest in children [1,38]. This result is expected, considering that the route of transmission for PVB19 is most efficient among children in educational facilities.

Even though the majority of samples were collected from hospitalized patients, there was a higher percentage of positive PVB19 IgM among the patients in ambulatory care (9.26%) compared to hospitalized patients (8.10%). This is likely attributable to the usually mild clinical course of PVB19 infections, allowing immunocompetent patients to adequately manage their symptoms through self-care treatment at home. Individuals with severe anemia may necessitate hospitalization and blood transfusion therapy, while immunocompromised patients, who are at a higher risk of developing complications, may receive antibodies to treat the infection [39]. Also, it is possible that lower percentage of positive PVB19 IgM samples from inpatients is, at least partially, due to the fact that this testing is not always done because PVB19 is suspected, but rather for screening purposes to several viruses, when clinical presentation is not clear.

Furthermore, sex-based disparities in PVB19 seroprevalence were noted, with females exhibiting a significantly higher prevalence (51.90%) compared to males (42.50%). Analogous female to male variations of seroprevalence were observed in the German general population (73.30% vs. 70.90%) [18] and Turkish blood donors (65.40% vs. 57.20%) [24], while Croatian [10] and Polish residents [36] displayed equitable burdens between sexes. Regarding the level of IgM antibodies, there was no statistically significant difference between sexes (9.80% for women vs. 6.96% for men). This aligns with the results of a study conducted in Turkey where the seropositivity rate for IgM was 11.10%, with no significant difference between males and females [40]. The higher prevalence in women might be attributed, in part, to their greater likelihood of caring for young children, potentially enhancing virus exposure [18]. However, these variations cannot be fully explained by caregiving patterns and may stem from differences in health status, patient age, and potential variations in test sensitivity.

We also monitored the monthly distribution of acute PVB19 infections throughout the calendar year with the aim of identifying any seasonal patterns in infection occurrence. During the period under surveillance (2008–2023), the highest percentages of positive IgM samples, 13.51%, 11.98%, and 11.10%, were reported in May, June, and July, respectively. Similar results were obtained in an eight-year study conducted in Ireland, with most cases diagnosed between March and July [41], and in a study conducted in Israel, with a major peak of acute cases during June [17]. Our study recorded the highest frequency of IgM-positive cases during the late spring and summer, potentially as a result of different climatic conditions. Additionally, the fact that PVB19 infection is not a mandatory notifiable disease in Serbia raises the possibility that the actual number of PVB19 cases may substantially exceed the number of registered cases. Furthermore, the extent to which our data are representative of the true distribution of acute PVB19 infections in the general population is debatable due to a potential testing bias favoring certain high-risk population groups, particularly young children, pregnant women, and the immunocompromised. Although the incidence of PVB19 infections exhibits considerable seasonal and year-to-year variations, a clearly visible pattern remains elusive. Moreover, upon further analysis of the yearly distribution of positive IgM samples, we observed that the highest percentage was reported in 2011 (18.70%), followed by 2012 (17.10%) and 2013 (15.40%). According to the literature data, a larger number of PVB19 cases in the form of epidemics typically occurs every 3–5 years, with more cases registered during the winter and spring seasons. This suggests that environmental factors such as weather and geographical conditions probably affect the PVB19 prevalence and seasonal infection rates. However, some studies have reported slightly different data [40,41].

Concerning clinical symptom manifestations caused by PVB19, we found that dermatological presentations were predominant, although other manifestations, including rheumatological and hematological, were not uncommon. Accordingly, our results showed that the highest percentage of acutely ill patients (42.86%) was observed among those diagnosed with erythema infectiosum, followed by transplant recipients (21.42%) and women experiencing miscarriage (20%). These outcomes align with expectations, given that erythema infectiosum is the most common clinical presentation of PVB19 infection in immunocompetent individuals. Transplant recipients exhibit heightened susceptibility to PVB19 infections due to the increased use of induction therapy to prevent early graft rejection [42]. Huang et al.’s 2022 study found a 10.17% infection rate of PVB19 after kidney transplantation [43]. Among PVB19-infected patients, individual variations in antibody production time and titers were noted, with PVB19 IgG/IgM generally appearing 2–3 weeks after viremia. Several factors, including the timing of viremia, high viral load, persistence, and resistance to virus inactivation procedures, contributed to the risk of transmission through blood, blood products, bone marrow, and solid-organ transplantation [44,45,46,47]. PVB19 infections have been reported as complications following both solid-organ and hematopoietic stem cell transplantations. The most common manifestation of PVB19 infection in transplanted patients is anemia, although the virus has also been linked to a range of other, potentially serious, complications, including hepatitis, pneumonia, myocarditis, and allograft dysfunction [48]. In this study, an acute PVB19 infection was proven in 12.50% of patients diagnosed with arthralgia/arthritis. A review of the literature data indicates that approximately one-third of adults with a PVB19 infection experience acute joint symptoms, a higher prevalence than what was observed in children [49,50]. Nevertheless, individuals of all ages can be affected by chronic and prolonged rheumatic symptoms, including children [1]. In a study conducted in Turkey, positive PVB19 IgM and IgG antibodies were detected in 10.20% of patients with diseases of the musculoskeletal and connective tissues [40]. PVB19 infections can also cause serious cardiac complications, particularly in children (i.e., pediatric myocarditis), which can take a severe clinical course, leading to high morbidity and mortality rates, including the need for a heart transplant surgery [51]. In line with this, acute PVB19 infection was detected in 6.25% of patients diagnosed with myocarditis.

PVB19 seroprevalence among pregnant women may vary geographically and is influenced by factors such as population immunity, healthcare practices, and socioeconomic conditions. In our study, samples from 411 pregnant women were analyzed, revealing a prevalence of immunity due to previous exposure to PVB19 of 53.04%. This prevalence ranged from 46.30% in the youngest group (16–25 years old) to 55.21% in the 36–45 years old age group. These data are consistent with global estimates, which suggest that approximately 30–50% of pregnant women lack immunity to PVB19, increasing the likelihood of infection and vertical transmission to the fetus. On the other hand, the incidence of acute PVB19 infection during pregnancy has been found to range from 1% to 5%, exceeding 10% during epidemic periods [16,26]. Seroprevalence in our study was slightly higher than the aforementioned results, i.e., approximately 10.22% of pregnant women showed serological evidence of an acute PVB19 infection at the time of testing. Virus transmission is facilitated by frequent and close physical contact, and since children are the likely source of infection for most adults, there may be increased risk for mothers through family contact with their own children who, in turn, acquire the infection in children’s day-care centers, schools, or playgrounds. Additionally, daycare workers, school teachers, and healthcare workers may be at special risk of infection during the early months of pregnancy. These types of observations have been reported in studies carried out in Germany and Canada [18,52]. While infections caused by PVB19 are generally mild in healthy individuals, they can pose a significant risk to pregnant women and their developing fetuses. Infections during pregnancy may result in complications such as fetal hydrops, anemia, and non-immune hydrops, especially when the infection occurs in the first half of gestation. In our study, serological analysis of women who experienced miscarriages revealed that 20% of them had acute PVB19 infection. Additionally, 44% of these women exhibited IgG antibodies (from previous infection), while 36% were immunologically naïve. These findings underscore the importance of assessing the immune status of PVB19 in pregnant women to closely monitor those with acute infections and manage potential complications.

Furthermore, the serological status of women in the reproductive age group from our region closely mirrors the profile of pregnant women. The immunological status of non-pregnant women of reproductive age (14–45 years old) indicates an overall prevalence of 51.12%, ranging from 52.55% in those aged 26–35 years to 55.91% in those aged 36–45 years. These findings align with other studies reporting that 50–75% of women of reproductive age have already developed immunity to PVB19 [16,53]. The percentage of serologically tested women of reproductive age with sufficient levels of IgG antibodies to provide protection against a PVB19 infection during pregnancy is of great importance. Our study showed that as many as 36.20% of women of reproductive age have not been infected with PVB19 and are thus at risk of developing an infection during a future pregnancy. The results of the research coincide with the findings of a Dutch study, in which the percentage of women of reproductive age without a prior PVB19 infection was 35–45% [54]. Similar results were obtained in studies conducted in several European countries, which determined that the percentage of women of reproductive age who did not have protective IgG antibodies was 43.50% in Finland, 39.90% in Italy, 38.10% in England and Wales, 36.80% in Poland, and 26% in Belgium [26].

Our study has several limitations. First, although we used two well-validated serological assays for the determination of type-specific antibodies (PVB19 IgM and IgG), potential variations in the sensitivity and specificity of these assays may have influenced the estimated seroprevalence. Second, a subset of tested samples exhibited borderline results, yet an alternative assay was not administered to elucidate these outcomes. Consequently, all patients with ELISA borderline results, where diagnosis remained unresolved, were excluded from the study. We believe that the number of equivocal samples was sufficiently negligible to minimally impact the study’s conclusions. Third, the total sample was not standardized; thus, a small number of samples were registered in some age categories (e.g., younger than one year, 5–9 years old, and ≥60 years), which might have influenced the reported prevalence. Moreover, it should be acknowledged that the comparison of our results with studies conducted in other geographical regions should be interpreted in the context of those studies’ designs. Fourth, taking into account the absence of a PVB19 case definition, the reported prevalence is the result of a random sample based on the indications of physicians in the system of primary and higher levels of health care. Next, the data collection was confined to basic socio-demographic variables, lacking inclusion of other behavioral data that could facilitate the assessment of the significance of specific risk factors for PVB19 within our country. Given that all participants currently reside in Vojvodina, insight into their place of birth in Serbia was unavailable. Additionally, information regarding the month of pregnancy was not obtained. A notable strength of this study resides in the extensive sample size sourced from the general population, spanning an age range from a few months to 90 years, encompassing diverse age categories. Furthermore, the study included participants from outpatient settings as well as hospitals. Such comprehensive datasets remain scarce in most southeastern European countries, as previous seroepidemiological studies predominantly focused on cohorts with restricted age or other demographic and clinical parameters. To the best of our knowledge, this survey stands as the sole examination of PVB19 seroprevalence in the general population of Serbia since the 2008 study conducted by Milosevic et al. [38].

## 5. Conclusions

As the current guidelines do not recommend routine exclusion of pregnant women from workplaces involving contact with children or patients with PVB19, it appears reasonable to provide mandatory screening of pregnant women for PVB19-specific IgG. It is important to emphasize that the characteristic rash (i.e., erythema infectious) typically appears about two weeks after exposure, possibly outside of the infectious period and coinciding with the emergence of IgM antibodies, meaning that the infectious period of the disease precedes the onset of visible symptoms. In addition, PVB19 testing is not incorporated into routine preconception or prenatal screening protocols, despite PVB19’s recognized feto-tropic potential and the possibility of fetal loss, resulting in a potential delay in diagnosis and management of PVB19-induced pregnancy complications. Also, precise etiological diagnosis of any given rash is important not just for disease treatment but for distinction between PVB19 and suspected measles and rubella outbreak(s). In the absence of commercially available PVB19 vaccine, the infection prevention relies solely on robust hygiene practices and widespread awareness of the infection’s consequences, diagnostic methods, and preventive measures, which further highlights the importance of raising awareness about PVB19 among both healthcare workers and the general population.

## Figures and Tables

**Table 1:** General characteristics of the study population and seroprevalence of PVB19 IgM antibodies, Vojvodina, Serbia, 2008–2023.

IgM	Total Samples Tested, n	Total IgM Positive Samples		Ambulatory Patients’ Samples			Hospitalized Patients’ Samples			*p*-Value
		n	% (95% CI)	Tested	IgM Positive, n	% (95% CI)	Tested	IgM Positive, n	% (95% CI)	
**Total**	8692	753	8.66 (8.08–9.27)	4200	389	9.26 (8.40–10.18)	4492	364	8.10 (7.32–8.94)	0.055
**Sex, n (%)**										
**male**	3477	242	6.96 (6.14–7.86)	1297	93	7.17 (5.83–8.71)	2180	149	6.83 (5.81–7.98)	0.707
**female**	5215	511	9.80 (9.00–10.64)	2903	296	10.20 (9.12–11.35)	2312	215	9.30 (8.15–10.56)	0.279
**Age category, n (%)**										
**<1**	238	14	5.88 (3.25–9.67)	31	1	3.23 (0.08–16.70)	207	13	6.28 (3.39–10.50)	0.500
**1–4**	1015	92	9.06 (7.37–11.00)	204	25	12.25 (8.09–17.56)	811	67	8.26 (6.46–10.37)	0.076
**5–9**	721	72	9.99 (7.89–12.41)	213	24	11.27 (7.35–16.30)	508	48	9.45 (7.05–12.33)	0.457
**10–19**	1340	141	10.52 (8.93–12.29)	529	57	10.78 (8.26–13.73)	811	84	10.36 (8.35–12.66)	0.808
**20–39**	2850	265	9.30 (8.26–10.42)	1919	195	10.16 (8.85–11.60)	931	70	7.52 (5.91–9.40)	**0.023**
**40–59**	1710	130	7.60 (6.39–8.96)	964	73	7.57 (5.98–9.43)	746	57	7.64 (5.84–9.79)	0.958
**≥60**	818	39	4.77 (3.41–6.46)	340	14	4.12 (2.27–6.81)	478	25	5.23 (3.41–7.62)	0.462

Note: n = number of samples. In bold are significant results at *p* < 0.05.

**Table 2:** General characteristics of the study population and seroprevalence of PVB19 IgG antibodies, Vojvodina, Serbia, 2008–2023.

IgG	Total Samples Tested, n	Total IgG Positive Samples		Ambulatory Patients’ Samples			Hospitalized Patients’ Samples			*p*-Value
		n	% (95% CI)	Tested	IgG Positive, n	% (95% CI)	Tested	IgG Positive, n	% (95% CI)	
**Total**	7325	3529	48.18 (47.03–49.33)	3995	2195	54.94 (53.39–56.49)	3330	1334	40.06 (38.39–41.75)	**<0.001**
**Sex, n (%)**										
**male**	2892	1229	42.50 (40.69–44.32)	1219	591	48.48 (45.64–51.33)	1673	638	38.14 (35.80–40.51)	**<0.001**
**female**	4433	2300	51.88 (50.41–53.36)	2776	1604	57.78 (55.92–59.63)	1657	696	42.00 (39.61–44.42)	**<0.001**
**Age category, n (%)**										
**<1**	183	76	41.53 (34.31–49.03)	25	8	32.00 (14.95–53.50)	158	68	43.04 (35.20–51.14)	0.298
**1–4**	866	79	9.12 (7.29–11.24)	185	19	10.27 (6.30–15.57)	681	60	8.81 (6.79–11.19)	0.541
**5–9**	640	126	19.69 (16.67–22.98)	197	45	22.84 (17.18–29.35)	443	81	18.28 (14.79–22.20)	0.181
**10–19**	1174	505	43.02 (40.16–45.90)	492	239	48.58 (44.08–53.09)	682	266	39.00 (35.32–42.78)	**0.001**
**20–39**	2468	1461	59.20 (57.23–61.14)	1847	1097	59.39 (57.11–61.64)	621	364	58.62 (54.63–62.52)	0.733
**40–59**	1399	916	65.48 (62.92–67.97)	922	590	63.99 (60.80–67.10)	477	326	68.34 (63.96–72.50)	0.105
**≥60**	595	366	61.51 (57.47–65.44)	327	197	60.24 (54.71–65.59)	268	169	63.06 (56.98–68.85)	0.483

Note: n = number of samples. In bold are significant results at *p* < 0.05.

**Table 3:** Seroprevalence of PVB19 IgG/IgM according to sex and age category in Vojvodina, Serbia, 2008–2023.

		IgM Seropositive, n (%)	IgM Seronegative, n (%)	*p*-Value
**Males**	children and adolescents	147 (8.62)	1558 (91.38)	**0.001**
	adults	90 (6.28)	1342 (93.72)	
	elderly	5 (2.58)	189 (97.42)	
	total	242 (7.27)	3089 (92.73)	NA
**Females**	children and adolescents	172 (12.14)	1245 (87.86)	**0.008**
	adults	321 (9.91)	2919 (90.09)	
	elderly	18 (6.57)	256 (93.43)	
	total	511 (10.36)	4420 (89.64)	NA
**Total**	children and adolescents	319 (10.22)	2803 (89.78)	**0.001**
	adults	411 (8.80)	4261 (91.20)	
	elderly	23 (4.91)	445 (95.09)	
	total	753 (9.11)	7509 (90.89)	NA
		**IgG Seropositive, n (%)**	**IgG Seronegative, n (%)**	***p*-value**
**Males**	children and adolescents ^#^	347 (24.79)	1053 (75.21)	**<0.001**
	adults	738 (62.86)	436 (37.14)	
	elderly	98 (71.01)	40 (28.99)	
	total	1183 (43.62)	1529 (56.38)	NA
**Females**	children and adolescents ^#^	363 (29.71)	859 (70.29)	**<0.001**
	adults	1781 (62.58)	1065 (37.42)	
	elderly	126 (64.62)	69 (35.38)	
	total	2270 (53.25)	1993 (46.75)	NA
**Total**	children and adolescents ^#^	710 (27.08)	1912 (72.92)	**<0.001**
	adults	2519 (62.66)	1501 (37.34)	
	elderly	224 (67.27)	109 (32.73)	
	total	3453 (49.51)	3522 (50.49)	NA

Note: A total of 430 (4.95%) samples tested for IgM and 167 (2.34%) samples tested for IgG had an equivalent result, in accordance with the manufacturer’s recommendations, and were excluded from further analyses. Age categories: children and adolescents (0–19 years old), adults (20–64), and elderly (≥65 years old). ^#^ for the IgG analysis, the children and adolescent’s category includes 1–19 years old patients, since in <1-year-old patients IgG might also have derived from mothers by passing transplacentary. n = number of samples. In bold are significant results at *p* < 0.05.

**Table 4:** Serological results for the PVB19 in Vojvodina, Serbia, 2008–2023.

Serological Results	n	%	95% Confidence Interval
IgM+/IgG−	247	3.66	3.23–4.14
IgM+/IgG+	366	5.43	4.90–5.99
IgM−/IgG+	2942	43.63	42.44–44.82
IgM−/IgG−	3188	47.28	46.08–48.48
**Total**	6743	100	100

**Table 5:** Prevalence of PVB19 immunological status by sex and age category in Vojvodina, Serbia, 2008–2023.

		Total, n (%)	Acute, n (%)	Immune, n (%)	Naive, n (%)	*p*-Value
**Males**	children and adolescents	1438 (100)	147 (10.22)	314 (21.84)	977 (67.94)	**<0.001**
	adults	1152 (100)	90 (7.81)	666 (57.81)	396 (34.38)	
	elderly	134 (100)	5 (3.73)	90 (67.16)	39 (29.10)	
	total	**2724 (100)**	242 (8.88)	1070 (39.28)	1412 (51.84)	NA
**Females**	children and adolescents	1232 (100)	172 (13.96)	283 (22.97)	777 (63.07)	**<0.001**
	adults	2730 (100)	321 (11.76)	1474 (53.99)	935 (34.25)	
	elderly	197 (100)	18 (9.14)	115 (58.38)	64 (32.49)	
	total	**4159 (100)**	511 (12.29)	1872 (45.01)	1776 (42.70)	NA
**Total**	children and adolescents	2670 (100)	319 (11.95)	597 (22.36)	1754 (65.69)	**<0.001**
	adults	3882 (100)	411 (10.59)	2140 (55.13)	1331 (34.29)	
	elderly	331 (100)	23 (6.95)	205 (61.93)	103 (31.12)	
	total	**6883 (100)**	753 (10.94)	2942 (42.74)	3188 (46.32)	NA

Note: children and adolescents (0–19 years old), adults (20–64), and the elderly (≥65 years old). n = number of samples. In bold are significant results at *p* < 0.05.

**Table 6:** Prevalence of PVB19 immunological status by age category in the sample of pregnant women, Vojvodina, Serbia, 2008–2023.

	Acute			Immune			Naïve			Total
Age Category	n (%)	OR (95% CI)	*p*-Value	n (%)	OR (95% CI)	*p*-Value	n (%)	OR (95% CI)	*p*-Value	n (%)
14–25	2 (3.70)	ref.		25 (46.30)	ref.		27 (50.00)	ref.		54 (100)
26–35	28 (10.73)	3.12 (0.72–13.53)	0.128	140 (53.64)	1.34 (0.75–2.42)	0.326	93 (35.63)	0.55 (0.31–1.00)	0.05	261 (100)
36–45	12 (12.50)	3.71 (0.80–17.26)	0.094	53 (55.21)	1.43 (0.73–2.79)	0.295	31 (32.29)	**0.48 (0.24–0.94)**	**0.034**	96 (100)
**Total**	42 (10.22)	NA	NA	218 (53.04)	NA	NA	151 (36.74)	NA	NA	411 (100)

Note: n = number of samples. In bold are significant results at *p* < 0.05.

**Table 7:** Prevalence of PVB19 immunological status by age category in the sample of non-pregnant women of generative age (14–45 years old), Vojvodina, Serbia, 2008–2023.

	Acute			Immune			Naïve			Total
Age Category	n (%)	OR (95% CI)	*p*-Value	n (%)	OR (95% CI)	*p*-Value	n (%)	OR (95% CI)	*p*-Value	n (%)
14–25	69 (11.90)	ref.		255 (43.97)	ref.		256 (44.14)	ref.		580 (100)
26–35	88 (12.81)	1.09 (0.78–1.52)	0.623	361 (52.55)	**1.41 (1.13–1.76)**	**0.002**	238 (34.64)	**0.67 (0.53–0.84)**	**0.001**	687 (100)
36–45	87 (13.18)	1.12 (0.80–1.58)	0.496	369 (55.91)	**1.62 (1.29–2.02)**	**<0.001**	204 (30.91)	**0.57 (0.45–0.71)**	**<0.001**	660 (100)
**Total**	244 (12.66)	NA	NA	985 (51.12)	NA	NA	698 (36.22)	NA	NA	1927 (100)

Note: In bold are significant results at *p* < 0.05.

**Table 8:** Uni- and multi-variate logistic regression analysis for factors associated with PVB19 acute infection status in Vojvodina, Serbia, 2008–2023.

	OR	95% CI	*p*-Value	aOR	95% CI	*p*-Value
**Sex**						
male	ref.	ref.	ref.	-	-	-
female	**1.44**	**1.22–1.69**	**<0.001**	-	-	-
**Women**						
generative age non-pregnant	ref.	ref.	ref.	ref.	ref.	ref.
pregnant women	0.81	0.57–1.14	0.229	0.81	0.57–1.14	0.223
**Age, cont. (one-year increase)**	**0.99**	**0.99–0.99**	**0.001**	-	-	-
**Age category**						
children and adolescents	ref.	ref.	ref.	ref.	ref.	ref.
adults	0.87	0.75–1.02	0.086	0.99	0.75–1.34	0.995
elderly	**0.55**	**0.35–0.85**	**0.008**	0.85	0.43–1.66	0.631
**School-age category**						
<1	ref.	ref.	ref.	ref.	ref.	ref.
1–3	1.43	0.79–2.60	0.239	1.52	0.82–2.80	0.181
4–6	1.49	0.80–2.78	0.209	1.7	0.83–3.48	0.148
7–14	1.74	0.97–3.10	0.062	2.3	0.89–5.94	0.084
15–19	1.39	0.76–2.55	0.288	2.16	0.56–8.33	0.264
**Season when testing was performed**						
spring	ref.	ref.	ref.	ref.	ref.	ref.
summer	0.94	0.77–1.15	0.532	0.94	0.77–1.15	0.568
autumn	**0.61**	**0.49–0.76**	**<0.001**	**0.61**	**0.49–0.76**	**<0.001**
winter	0.82	0.66–1.01	0.057	0.81	0.66–1.01	0.056

Note: OR = odds ratio; aOR = adjusted OR for age (cont.) and sex of the patients; 95% CI = 95% confidence interval; ref. = reference group. In bold are significant results at *p* < 0.05.

## Data Availability

The data that support the findings of this study are available from the corresponding author upon reasonable request.

## Supplementary Materials

The following supporting information can be downloaded at: https://www.mdpi.com/article/10.3390/v16020180/s1, Figure S1. Number of tested samples and percent of positive PVB19 IgM samples across four meteorological seasons in Vojvodina, Serbia, 2008–2023; Figure S2. Percent of PVB19 IgM tested samples in Vojvodina, Serbia, 2008–2023.
