# Supplementary material for: Seroepidemiology of Human Parvovirus B19 Infection among the Population of Vojvodina, Serbia, over a 16-Year Period (2008–2023)
Source: Viruses. 2024 Jan 25;16(2):180. doi: 10.3390/v16020180 (PMC10893261; doi:10.3390/v16020180)
Supplement: Supplementary file 1 [file viruses-16-00180-s001.zip › viruses-2837112-supplementary.pdf]

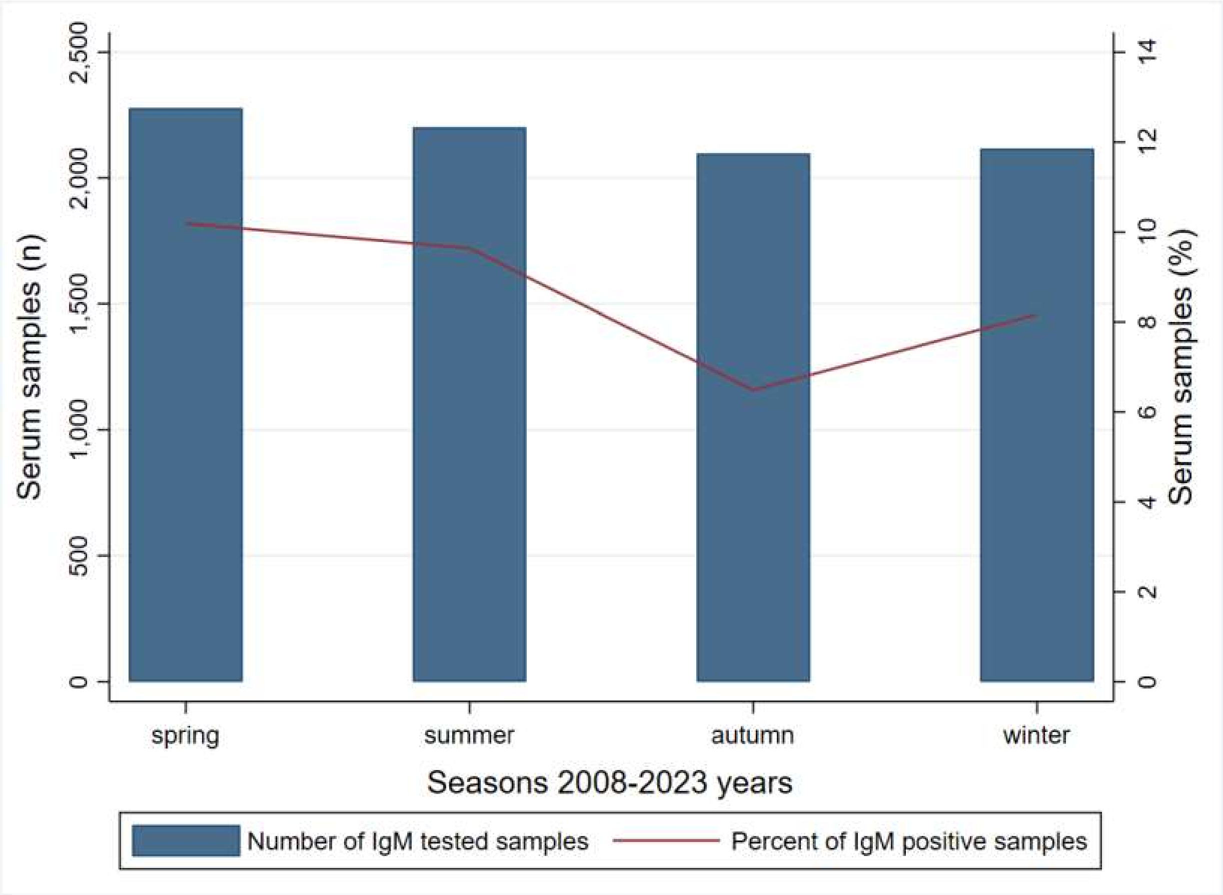

Figure S1. Number of tested samples and percent of positive PVB19 IgM samples across four meteorological seasons in Vojvodina, Serbia, 2008-2023.

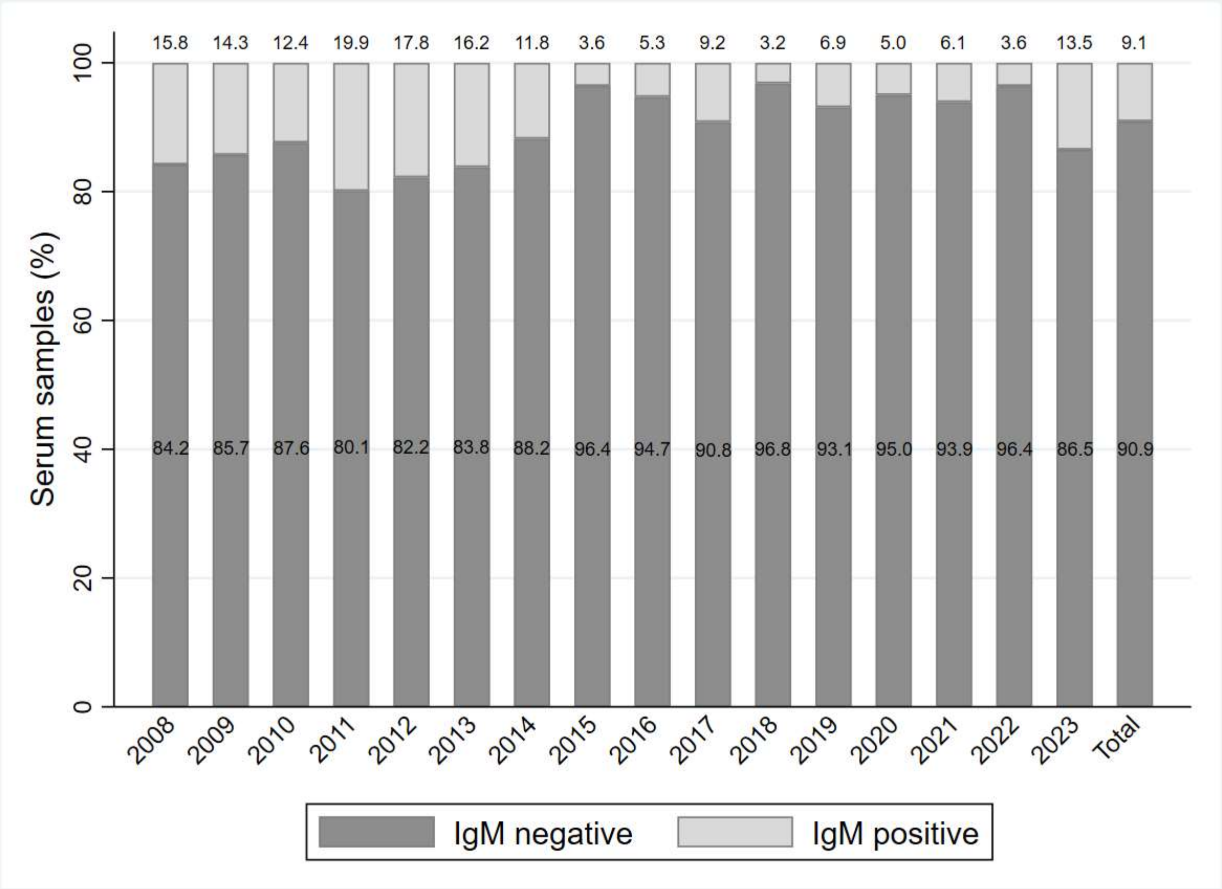

Figure S2. Percent of PVB19 IgM tested samples in Vojvodina, Serbia, 2008-2023.
